# Transgenerational Trauma and Mental Health Needs among Armenian Genocide Descendants

**DOI:** 10.3390/ijerph181910554

**Published:** 2021-10-08

**Authors:** Alissa Der Sarkissian, Jill D. Sharkey

**Affiliations:** Department of Clinical, Counseling, and School Psychology, Gevirtz Graduate School of Education, University of California, Santa Barbara, CA 93117, USA; jsharkey@ucsb.edu

**Keywords:** Armenian mental health, transgenerational trauma, genocide survivor, refugee, multicultural school psychology, trauma-informed

## Abstract

The trauma of a genocide can be transmitted to subsequent generations though familial mental health, sociopolitical trauma, and cultural narratives, thereby impacting mental health and well-being. Understanding specific mechanisms that are unique to each ethnic group impacted by genocide illuminates cultural, sociopolitical, and individual factors related to the transmission. For the Armenian community, the unresolved historical loss of the Armenian Genocide of 1915, with the threat of acculturation for such a large diasporic population, a continued denial by the perpetrators, as well as subsequent generations’ refugee experiences, may further exasperate the impact of transgenerational trauma from the genocide. This literature review explores the mental health needs of Armenian youth in the current sociopolitical context and provides implications for how schools and communities may use this knowledge to inform supports that center Armenian community healing. Future directions for research are also discussed.

## 1. Introduction

Despite international attempts to prevent future genocides through the work of the UN Convention on the Prevention and Punishment of the Crime of Genocide, perpetrators continue to commit genocide, such as one of the most recent cases against the Rohingya in Myanmar [1]. Given the modern devastation caused by genocidal acts across the world and the continued impact of historical genocides, it is critical to better address the mental health impact on survivors and their descendants. While most of the research on the psychological impact of genocide focuses on direct survivors, research on if and how trauma symptoms are passed down to subsequent generations is mixed. The following paper centers the sociopolitical and cultural factors crucial to understanding a community of genocide descendants and integrates them with existing guidance and gaps in the literature, using Armenian descendants as a case example.

School and communities have an increasing role in supporting the health and well-being, rather than the control and punishment, of students and community members. Schools are no longer simply institutions of education, but also take on the responsibility to assess, diagnose, and provide appropriate interventions or referrals to students in need. Mental health professionals trained in multicultural psychology are responsible to acquire knowledge of the sociopolitical context of the communities they serve and to learn the appropriate idioms of distress. Without this knowledge, they would not be able to properly identify youth in need or use the appropriate terminology to communicate effective treatment. Some school psychologists suggest that trauma-informed interventions are needed to respond appropriately to students with trauma exposure [2]. Ginwright [3] proposes that psychologists build from a trauma-informed approach to adopt a healing-centered engagement plan, in which they acknowledge and center strengths. For example, a community psychologist who uses a trauma-informed approach may contextualize a youth’s behavioral issues as rooted in their experiences from homelessness and involvement in the foster system. While this viewpoint may build empathy and help address some of the youth’s mental health needs, it also places the relationship in a strictly deficit-based lens. Through a healing-centered framework, mental health professionals can create a more holistic expression of care that incorporates culture, spirituality, and collective healing.

## 2. Methods

This review of the literature was guided by the following research questions:

1. How do Armenian Americans experience transgenerational trauma?
2. How does sociopolitical context, such as genocide denial and subsequent community trauma, influence how transgenerational trauma of a genocide is transmitted?
3. What are the cultural and community-based considerations for researchers, mental health practitioners, and community leaders working with Armenian American populations?

To answer these questions, a review of relevant literature was conducted using the following databases: Google Scholar, PsycInfo, and PubMed. A systematic narrative review technique was used to synthesize and critically evaluate the literature. Given the limited research available on Armenian mental health, the risk of bias in study inclusion was limited. Each search used a combination of key search terms, including: “genocide descendant mental health”, “transgenerational trauma”, “historical loss”, Armenian mental health”, and “Middle Eastern mental health.” Studies were excluded if they did not use validated measures or were in languages other than English.

## 3. Transgenerational Trauma among Genocide Survivors

Transgenerational trauma is the transference of emotional, physical, mental, spiritual, and social distress that, when untreated, can be compounded within and across generations among either an individual family or a community [4]. A meta-analysis examined the mental health impact of Holocaust survivors and their descendants and included 55 studies with children of survivors and seven studies with grandchildren of survivors [5]. They found higher levels of depression, anxiety, and paranoia for children and grandchildren of survivors as compared to various types of control groups, including descendants of American-born Jewish grandparents and pre- or post-war non-survivor immigrants. The results suggest that symptoms of unresolved trauma can be passed down to descendants directly, through symptoms such as familial conflict or parental substance use, or indirectly, such as through epigenetic transmission due to changes in how genes are expressed [6,7]. Despite this evidence, some researchers posit that increased mental health needs among Holocaust descendants stems from the stress of immigration and acculturation rather than from passed down and inherited trauma [8]. While qualitative research suggests that second- and third- generation descendants of genocide survivors experience transgenerational trauma and that mental health symptoms are passed down directly or indirectly, meta-analyses of quantitative studies with descendants conclude a lack of evidence for this type of transmission [9]. These meta-analyses (e.g., Sagi-Schwartz et al. [10]) solely compared grandchildren of Holocaust survivors to non-Holocaust control groups in terms of mental health outcomes. The results do not consider the full extent to which potential familial and societal factors impact whether and how trauma is inherited and passed on. For example, other ethnic groups who are genocide survivors do not have the same worldwide recognition and reparations that Holocaust survivors have benefited from, and which have promoted resilient growth and healing.

To parse apart the complex concept of transgenerational trauma, it is imperative to explore how different sociopolitical and familial contexts influence the outcomes of genocide survivors and their descendants. For example, second-generation Holocaust survivors reported higher levels of mental health issues if their mothers were 18 years old or younger during the genocide and survived alone [11]. A study on the descendants of the Tutsi genocide in Rwanda found that exposure to higher numbers of traumatic stressors during the war, levels of physical illness, and levels of social integration all predicted symptoms of PTSD among children of survivors [12]. These two findings exemplify the importance of considering specific familial experiences of trauma in predicting the level of impact to descendants. Alternatively, the level of identification with a historically victimized group can contribute to the descendants’ interpretation, and inheritance, of the trauma. For example, Wohl and Van Bavel [13] found that levels of ethnic identification with their Jewish background among Holocaust descendants were positively associated with post-traumatic stress disorder (PTSD) symptoms. In contrast, ethnic identification was *negatively* associated with PTSD among the non-genocide-descendant Jewish comparison group. The authors suggest that these results indicate a possible mediating relation between ethnic identification and transgenerational trauma. However, the results could also indicate that families who experienced a greater toll from a genocide may exhibit stronger connection to their ethnic identity and also present with higher mental health symptomology among their descendants. Therefore, research needs to disentangle how and when descendants of genocide survivors continue to be impacted.

## 4. The Sociopolitical Context of the Armenian Genocide

The historical context of a genocide, such as global recognition, reparations, and further persecution from the perpetrators, could be an important consideration regarding the impact of a genocide on descendants and on members within the ethnic group in subsequent generations. For the Armenian Genocide, the modern denial of the genocide by the Turkish government and modern discrimination against Armenians in their lost homeland refuel the sense of ambiguous loss within the Armenian community [14,15]. These perceived ongoing injustices may prevent community healing [16]. The continued destruction of architectural and art history on stolen lands from the genocide, such as 2500 religious sites that have been destroyed and continue to be vandalized, flattened, or converted, deepen the wound of trauma for the descendants [17]. Brave Heart and Debruyn [18] named this phenomenon, “historical unresolved grief”, after noting the emotional reaction of the loss of lives, land, and cultural aspects among the American Indian and Alaskan Natives as similar to feelings associated with grief, such as guilt, anger, and helplessness.

Miller and Miller [19] found six major responses of survivors and descendants to the Armenian Genocide: (1) avoidance and repression; (2) outrage and anger; (3) revenge and restitution; (4) reconciliation and forgiveness; (5) resignation and despair; and (6) explanation and rationalization. Interestingly, second- and third-generation descendants of Armenian Genocide survivors were more likely to report anger and despair, while immediate survivors reported more feelings of avoidance, repression, and desire for forgiveness [19]. In their interviews, survivors linked the denial of the genocide as the most important impediment to their reconciliation. Importantly, survivors shared a theme of powerlessness in the current political context, centered around lack of recognition, as directly connected to the dehumanization and degradation they faced during the genocide. Since current descendants respond most with anger as they continue to suffer the consequences and bear the responsibility of the genocide, it can be suggested that the lack of recognition and reparations has a long-lasting impact on the collective psyche of the Armenian community.

After the genocide, the majority of Armenian families settled in Lebanon, Iran, and Syria [20,21]. For example, many refugees settled in Bourj Hammoud in Beirut, Lebanon, a town that became known as the Armenian quarter. However, as each of these countries struggled in sociopolitical turmoil from the 1970s to today, many of these families then experienced a second and sometimes third wave of refugee displacements [21,22]. Therefore, the Armenian American community constitutes multicultural families who often come from lineages of two to four generations of refugee experiences. Multiple generations of the family may have experienced similar types of intergenerational and transgenerational trauma. In this context, the experience of acculturation within an Armenian household can be particularly interesting to entangle, in that the “home culture” can include the food, music, customs, and values of multiple cultures.

While acculturation to the host culture may help some immigrants find a sense of belonging in their new homeland, others may sense deculturation, in which the immigrant feels loss of cultural identity and alienation [23]. Clifford [24] hypothesized that “peoples whose sense of identity is centrally defined by collective histories of displacement and violent loss cannot be ‘cured’ by merging into a new national community” (p. 307), especially within assimilationist national ideologies such as the United States. To deculturate can feel as though the descendant is further perpetrating the last stage of their own ethnic group’s genocide. To describe this sense of cultural loss, Eisenbruch defined cultural bereavement as: “the experience of the uprooted person—or group—resulting from loss of social structures, cultural values and self-identity… who suffers feelings of guilt over abandoning culture and homeland, feels pain if memories of the past begin to fade, but finds constant images of the past (including traumatic images) intruding into daily life, yearns to complete obligations to the dead, and feels stricken by anxieties, morbid thoughts, and anger that mar the ability to get on with daily life” [25] (p. 3).

Bhugra and Becker [26] note the importance of cultural bereavement to the mental health of various refugee groups, particularly those who migrate to individualistic societies from collectivistic societies. Similarly, Bâ [27] explored a similar experience regarding cultural bereavement among Bosnian genocide survivors. Given the history of genocide and multiple generations of displacement, Armenians have generally maintained a strong ethnic identity and an agenda to acculturate (adapt to the mainstream culture) quickly and assimilate (losing the home culture) slowly [28]. As William Saroyan said:

I should like to see any power of the world destroy this race, this small tribe of unimportant people, whose wars have all been and fought and lost, whose structures have crumbled, literature is unread, music is unheard, and prayers are no more answered. Go ahead, destroy Armenia. See if you can do it. Send them into the desert without bread or water. Burn their homes and churches. Then see if they will not laugh, sing and pray again. For when two of them meet anywhere in the world, see if they will not create a New Armenia [29] (p. 438).

This quote, famous among Armenians, exemplifies the group’s assigned importance on ethnic identity and community connectedness. Additionally, it connects the perceived fear of acculturation to the community’s resiliency against the genocide and other historical traumatic events, such as colonization and war. The Armenian diaspora is three times larger than the population living in Armenia at present. As the language of Western Armenian continues to lose speakers due to continued genocide and assimilation, it is now officially recognized as a “definitely endangered language” [30]. In this context, Armenians feel a sense of responsibility to maintain cultural and linguistic roots, and, therefore, collectively may feel the same sense of cultural bereavement. Among Armenians, this fear for loss of culture or “becoming White” has been colloquially named the “սպիտակ ջարդ” or the “սպիտակ ցեղասպանություն”, which translates to the “White break” or the “White Genocide” [31]. This term refers to the loss of Armenian culture while dispersed descendants assimilate in the diaspora that reside in Western countries. Additionally, descendants of the Armenian genocide report themes within their family system of needing to “super-achieve” to compensate for these familial losses [32]. Therefore, Armenian American youth and young adults may feel tension in choosing between the two cultures when the American majority expects them to assimilate and become “White” or American to succeed, while their Armenian community calls for a resistance to acculturation.

The sociopolitical factors of the genocide also contribute to the disparities that exist within the psychological research on various genocide victim groups [9]. As compared to over 1300 studies that have been published regarding the psychological ramifications of the Holocaust for survivors, there are fewer than 15 studies regarding the psychological consequences of the Armenian Genocide, with only one study that is of a truly empirical and quantitative nature [33]. Kay [33] proposed multiple reasons for these disparities, which included historical factors (including the occurrence of the Armenian Genocide before the “psychologized world”), political factors (Soviet Union and Turkey’s suppression of Armenian cultural identity and nationalistic activism), social factors (survivors as orphans; descendants did not have the economic means to pursue anything beyond basic survival), and professional factors (lack of Armenian representation in mental health fields). However, inclusion of the Armenian American experience within psychological knowledge is critical to help mental health providers support the health and wellbeing of the Armenian American community.

## 5. Mental Health of Armenian Americans

Although there are more than one million Armenians living in the United States (estimations are inexact given that Southwest Asian and North African groups are not counted as a racial group in the Census and many Armenians emigrated from various other countries [e.g., Iran and Lebanon] to the United States), the field of psychology has not invested in adequate research to inform effective mental health treatment and behavioral wellness services with Armenian Americans. Ninety percent of the Armenian population in the United States were offspring of genocide survivors [34]. Even 100 years after the Armenian Genocide, collective trauma continues to impact the mental health of Armenian people. Survivors of the 1988 earthquake in Armenia commonly reported nightmares with images related not only to the earthquake, but to the Armenian Genocide that their ancestors had experienced [35]. These connections illustrate the noted similarities between both of these instances of collective trauma, including mass deaths, disruption of community connection, and disconnection from the motherland. Additionally, the earthquake survivors’ connection to the genocide exemplifies how Armenians contextualize their modern experiences through the genocide. Karenian et al. [36] found that 35.7% of Armenian Americans report various symptoms of trauma, with the most severe impact reported among women, the elderly, and those with more familial impact of the genocide. Some participants reported survivor guilt, while others reported pain connected to injustices, such as discrimination, prejudice, and lack of recognition. Additionally, participants with more intense traumatic experiences reported closer ties to the community. Thus, there is evidence that Armenian Americans experience symptoms of trauma related to the Armenian Genocide and resulting displacement and violent loss of cultural artifacts. Additionally, contemporary contextual factors in the United States play a factor in the development of mental health concerns. Institutional discrimination, xenophobia, and a hostile American context towards SWANA communities can further exacerbate the impact of historical community-based trauma on both mental health and ethnic identity development [37]. For example, the backlash towards SWANA communities in the United States after September 11th, through increased surveillance and targeted hate crimes, triggered pervasive anxiety and alienation, a sense of victimization, and fears for safety [38] Transgenerational trauma is exacerbated by stigma and discrimination towards descendants in the context of modern society.

While some researchers report that descendants with direct familial exposure, meaning a direct parent or grandparent who survived the genocide, reported significantly higher depression and anxiety ratings compared to a non-direct familial exposure group [39], others found that perceived cultural impact of the genocide and Armenian ethnic identity, rather than perceived familial impact, were more likely to exhibit secondary trauma symptoms [40]. Given the strong values of both familism and collectivism, impacts may be influenced by both direct familial exposure and cultural identity. With such limited research on the descendants of the Armenian Genocide, the continued impact of the Armenian Genocide on the community’s mental health remains inadequately examined.

## 6. Cultural Factors on Mental Health Presentation

Another potential issue in determining the impact of transgenerational transmission of genocide trauma includes flawed clinical assessment measures of trauma-related distress among ethnic minority groups. Mental health research has historically utilized participants from Western, educated, industrialized, rich and democratic (WEIRD) societies, to make broad generalizations about mental health norms for the full extent of human diversity [41]. However, there may be essential differences in mental health presentation based on the expected forms of expressing distress in various cultures, such as somatization of mental health. For example, PTSD may be underdiagnosed among Cambodian genocide survivors because of cultural misunderstandings when a Western psychologist does not know the context of idioms of distress or acknowledge somatic symptoms, such as sweating, dizziness, and “weak heart” as culturally expected forms of psychological distress [42,43].

To desensitize trauma-related care and to better describe the interconnectedness of trauma to socio-economic and political contexts, practitioners have advocated for a re-conceptualization of post-traumatic distress to post-conflict eco-social adversity [44]. One suggested way to practice within this framework is to use and understand the ethnic group’s idioms of distress, which are “socially and culturally resonant means of experiencing and expressing distress” [45] (p. 405). For example, some Cambodian genocide survivors experience an idiom of distress called “baksbat”, which means “broken courage” and refers to a persistent fear that follows a distressing or life-threatening situation [42]. Cambodian respondents in a qualitative study contextualized baksbat within the socio-political context of feeling powerless in an authoritarian society, thinking too much about the events of the genocide, and fearing that a similar situation could occur again [46]. Therefore, a culturally competent practitioner would need to not only translate the idiom of distress, but also understand the social context of the term that communicates the expected symptomology and perceived cause of the symptom. Among Latinx groups, including Puerto Ricans and other Caribbean and Latin Americans, a group of idioms of distress called “ataques” are a response to a specific emotionally stressful event and include symptoms such as shouting uncontrollably, trembling, difficulty breathing, dizziness, and numbing or tingling sensations [47]. “Ataque” is interpreted as a means to communicate feelings of anger, frustration, or sadness and the need for support. However, the use of the phrase may vary depending on the individual’s specific cultural background, immigration history, and educational level [47]. Therefore, mental health practitioners should take care to not only learn the specific cultural norms around mental health but also encourage patients to communicate their interpretation and needs. That is, if a patient of an ethnic minority group is experiencing distress due to experiences of transgenerational trauma related to genocide, such as discrimination, secondary stress, and the systematic oppression of genocide denial, treatment may need to focus on building community strengths and resilience while acknowledging and addressing discrimination rather than approaching treatment from a more individualistic, Western lens.

Although there is no available research to date on idioms of distress among Armenians, some have been colloquially suggested. The “Komitas syndrome” is when a person involuntarily closes off parts of their ethnic identity associated with traumatic experience [14]. This syndrome is named after Komitas Vardapet, an Armenian priest and musicologist who was a genocide survivor and preserved ethnic music that would have been lost. He spent the last 13 years of his life in a psychiatric hospital and would not speak in his native tongue of Armenian about his past trauma or about his family or community. Gasparyan et al. [14] note that this same withdrawal is demonstrated in Armenian youth after a traumatic event. In these instances, just as maintaining ethnic identity is collectively recognized as resilience against the impact of the genocide, restraining from engagement with Armenian-ness has also been used as a defense against the impact of trauma, even when that part of their identity might help them to rehabilitate. Additionally, Armenians have demonstrated many idioms of distress that draw focus on the physical responses to pain and suffering. Therefore, delineating the specific idioms of distress that are used may help practitioners to learn how to support Armenian Americans and understand the connection between physical idioms of distress and the somatization of mental health needs.

Somatic complaints are physical pain symptoms that accompany mental health concerns, such as fatigue and stomachaches; these symptoms are associated with experiences of domestic violence, child abuse, and complex trauma [48]. Past research found that ethnic minority groups, such as Asian, Southwest Asian and North African (Southwest Asian and North African (SWANA) is a racial categorization of people to replace the term, “Middle Eastern,” given the problematic origins of the term due to Orientalist and colonial implications), and Latinx immigrants, experience more somatic symptoms to their mental health concerns when compared to WEIRD populations. Among Armenian Americans, Ayrapetyan [49] found a strong relation between depression and somatic or vegetative symptoms. While some scholars suggest that mental health stigma may make physical symptoms easier to discuss than emotional symptoms [50], relations between somatization and mental health stigma remain to be disentangled.

## 7. Future Directions of Research

This literature review delineates the current research investigating the impact of genocide on the descendants of survivors, particularly for Armenian descendants. Given the limited availability of research on this topic, future research that explores transgenerational trauma in the Armenian community through both qualitative and quantitative approaches will allow for further comparative perspectives and more guidance to mental health providers, school professionals, and community leaders. Further research should also consider incorporating diverse groups who have experienced genocide to build a cross-cultural understanding of genocide. Multiple familial, personal, and socio-political contexts play an important role in influencing the impact. Factors such as reparations, universal awareness of the genocide, and the level of global recognition of the trauma may impact the mental health of the impacted group and their ability to collectively heal from the historical loss.

Additionally, further research should investigate how a history of genocide, particularly for genocides that are politically and socially unresolved, may impact refugee populations’ perception of acculturation and acculturative stress. In particular, the experience of cultural bereavement should be examined among descendant groups with a large diasporic community, in which their acculturative stress is accompanied with fears of contributing to the extermination of their cultural or ethnic identity.

## 8. Conclusions

This paper highlights the unique considerations that require attention when working with Armenian American youth and the need for future research to better address these needs. The sociopolitical context of transgenerational trauma and multiple generations of refugee events impact the community’s experience and perception of acculturation and mental health. Current secondary trauma may play a role in students’ abilities to engage in school and in their academic performance. Schools and community mental health centers that support affected communities would benefit from implementing cultural considerations in treatment and case conceptualization.

Contemporary school- and community-based efforts to address mental health have adopted a preventive approach, as opposed to a reactive one, that prioritizes students’ needs and strengths while still considering the realistic limitations of available resources. For example, innovative restructuring of the school system can center consultation efforts to build an educational system that holds up youth of diverse backgrounds. A healthy collaborative relationship with families can both help families build trust towards the school system’s efforts to address their community’s needs and can support the school to build a system that will work for the students and their families [51]. Developing and encouraging attendance at school-and community-based events that uphold the histories of various communities can be a supportive way to encourage healing through community. For example, Glendale Unified in Los Angeles, with the largest Armenian population in the United States, offers the Armenian Genocide Remembrance Day, April 24th, as a day off, so that students can attend protests and commemorate with family [52]. Often, schools in the U.S. teach a White-centered version of history [53]. Instead, schools should include social studies course content that covers genocides and other important political strife across world history. This inclusion communicates to students that their histories are acknowledged, and their identities are important.

Therapy and assessment for mental health concerns can center transgenerational assessments and treatments such as genograms, which are family trees used in therapy to help the client understand transgenerational patterns of mental health, trauma, and resilience [14,54]. The clinician can also use the genogram to assess for the impact of transgenerational trauma and identify resiliency factors within the individual, familial, and community levels. As transgenerational trauma might impact these multiple levels of functioning, the therapist should enact a trauma-informed approach, even when therapy focuses on a topic that may not seem to be related to the trauma, such as identity. Guidance from the Adaption after Persecution and Trauma (ADAPT) model can allow the therapy to incorporate how community trauma may impact multiple areas of the client’s life, including attachment, feelings of hopelessness, identity development, and security [55]. For example, some Armenian community members have developed a mistrust for non-Armenians, especially those affiliated with large institutions, due to a history of genocide denial and forced displacement [56]. Therefore, clinicians who notice this mistrust should take more time to build rapport and build expectations. Lastly, clinicians can decolonize healing by centering the clients’ reported transgenerational resiliency factors that are already imbedded within the community as forms of healing, rather than trying to replace them with only Western approaches. For example, the Armenian community has heavily relied on values of resilience, community connectedness, and activism for centuries as forms of healing from transgenerational trauma [57]. Therefore, therapy can include these values, by incorporating group therapy in community-based sites and centering resiliency-based language. Including transgenerational, cultural, and sociopolitical considerations in the provision of mental health services may strengthen the efficacy of treatment for underrepresented groups.
